# Legacy of dignity: Words that are also mine

**DOI:** 10.1017/S1478951525100266

**Published:** 2025-06-20

**Authors:** Bianca Sakamoto Ribeiro Paiva

**Affiliations:** 1 Research Group on Palliative Care and Health-Related Quality of Life, Barretos Cancer Hospital, Barretos (SP), Brazil; 2 Institute of Education and Research, Barretos Cancer Hospital, Barretos (SP), Brazil.

Wherever I may be,
comes the active, compassionate, and wholehearted listening – the dignity therapist.
More than just a presence, it’s someone who is here to hear me,
to guide my words always in the direction of my living story.
That story which lives within me,
was mine and will be my legacy.
Memory dresses itself in noble recollections.
My life story, particularly the ones I want to remember,
the ones that matter most.
As a child, I was so happy, so whole,
I loved having coffee with milk
and smelling my mother’s cooking.
With great simplicity, I lived my childhood,
with early responsibility, I lived my adolescence;
and in adulthood, I found the love of my life.
Among questions stitched with affection,
memory keeps dressing itself in recollections.
The therapist gathers words like rare flowers
and builds a garden full of meaning.
They unveil the most important
(accomplishments)
from which I can feel proud.
I am filled with pride to be a complete mother,
leaving for my three children the very best of me
and shaping the best that exists within each of them.
There has not been, nor will there ever be,
a single day I don’t think of them.
Whether I remain in the earthly life or the transcendental.
Here, I will speak of love, of faith, of life,
of roles, of achievements,
of what I hope for the future of those I love.
I leave in this legacy document my presence,
who I was, who I wasn’t,
and who I am – Bianca.
And as I speak of what I’ve learned about
life and what I’d like to pass on to others,
my words echo softly,
and at the same time I understand:
this might be a farewell.
May they live with lightness,
but knowing that life often teaches us
in heavy and harsh ways.
May they be wholly present in all they do,
always steering away from the futile
and from (in)human cruelties.
And when they remember me,
may they smile,
because I was truly happy.
This legacy document eternalizes what matters to me.
These are the memories of my passage through this world,
Bianca the Daughter,
Bianca the Sister,
Bianca the Mother,
Bianca the Wife,
Bianca the Friend,
Bianca the Teacher,
Bianca the Researcher,
Bianca the Dignity Therapist,
and simply Bianca.

